# Dual or Not Dual?—Comparative Analysis of Fluorescence Microscopy-Based Approaches to Study Organelle Targeting Specificity of Nuclear-Encoded Plant Proteins

**DOI:** 10.3389/fpls.2018.01350

**Published:** 2018-09-19

**Authors:** Mayank Sharma, Bationa Bennewitz, Ralf Bernd Klösgen

**Affiliations:** Institute of Biology–Plant Physiology, Martin Luther University Halle-Wittenberg, Halle, Germany

**Keywords:** protein transport, dual targeting, nuclear-encoded proteins, endosymbiotic organelles, mitochondria, chloroplast

## Abstract

Plant cells are unique as they carry two organelles of endosymbiotic origin, namely mitochondria and chloroplasts (plastids) which have specific but partially overlapping functions, e. g., in energy and redox metabolism. Despite housing residual genomes of limited coding capacity, most of their proteins are encoded in the nucleus, synthesized by cytosolic ribosomes and need to be transported “back” into the respective target organelle. While transport is in most instances strictly monospecific, a group of proteins carries “ambiguous” transit peptides mediating transport into both, mitochondria and plastids. However, such dual targeting is often disputed due to variability in the results obtained from different experimental approaches. We have therefore compared and evaluated the most common methods established to study protein targeting into organelles within intact plant cells. All methods are based on fluorescent protein technology and live cell imaging. For our studies, we have selected four candidate proteins with proven dual targeting properties and analyzed their subcellular localization *in vivo* utilizing four different methods (particle bombardment, protoplast transformation, *Agrobacterium* infiltration, and transgenic plants). Though using identical expression constructs in all instances, a given candidate protein does not always show the same targeting specificity in all approaches, demonstrating that the choice of method is important, and depends very much on the question to be addressed.

## Introduction

As a consequence of the endosymbiotic gene transfer, the vast majority of proteins from mitochondria and chloroplasts are encoded in the nuclear genome, and synthesized in the cytosol of the eukaryotic plant cell (Martin and Herrmann, 1998; Bock and Timmis, 2008). Subsequent transport into the respective target organelle is mediated by N-terminal extensions, called presequences or transit peptides, which comprise all of the information for organelle targeting and transport. In most instances, this process is monospecific, i.e., a given protein is transported solely into a single type of organelle. However, a number of proteins exhibit so called dual targeting properties and can be imported into both, mitochondria and plastids (Peeters and Small, 2001). In many cases, such dual targeting is mediated by “ambiguous” transit peptides, which are thus capable of interacting with the protein import machineries of both endosymbiotic organelles (summarized in Carrie and Small, 2013). Current estimates assume that ~5% of the proteins from mitochondria and plastids possess dual targeting properties (Mitschke et al., 2009; Baudisch et al., 2014).

Dual targeting of a protein is often claimed based on a single experimental approach (e.g., Huang et al., 1990; Creissen et al., 1995; Silva-Filho, 1999; Masuda et al., 2003; Carrie et al., 2009). However, reevaluation of these findings with alternative assays sometimes leads to contradicting results (e.g., Chow et al., 1997; Lister et al., 2001). This holds particularly true if principally different experimental approaches are compared with each other, like transport experiments with isolated organelles, microscopy of transiently or stably transformed plant tissue, biochemical assays, or proteomics data (Tanz et al., 2013; Baudisch et al., 2014; Sharma et al., 2018). While in *in vitro* assays, such as *in organello* protein transport experiments, the import of authentic precursor proteins into purified intact organelles is studied, *in vivo* approaches usually rest on the transient or stable expression of chimeric reporter constructs in living cells. Both approaches have their pros and cons and each can address only specific, and often different, aspects of the transport process. For example, *in organello* experiments examine if a given precursor protein is, in principle, a suitable substrate for the organellar import machinery. However, this approach does not clarify if such transport will actually take place also in the presence of potentially regulatory cytosolic factors present in intact cells. On the other hand, *in vivo* approaches often analyze the subcellular localization of chimeric proteins comprised of the candidate protein fused to a fluorescent reporter. The potential influence of the heterologous reporter on the transport process, for example, as a result of its folding properties or of its position within the chimera (e.g., either downstream of the transport signal or at the very C-terminus of the candidate protein) has been described (e.g., Marques et al., 2004; Baudisch et al., 2014) but was not systematically analyzed.

Surprisingly, even in those instances where largely similar approaches are applied, like in the various *in vivo* assays employing fluorescent reporter proteins, deviating results are sometimes described. For example, AOX2 (alternative oxidase-2) of *Arabidopsis thaliana* was described by Saisho et al. (2001) to be targeted solely to mitochondria and by Fu et al. (2012) to be targeted to chloroplasts. Another protein, OhmT (3-Methyl-2-oxobutanoate hydroxy-methyl-transferase) of *A. thaliana*, showed with comparable *in vivo* approaches either monospecific transport into mitochondria (Ottenhof et al., 2004) or dual targeting to both endosymbiotic organelles (Baudisch et al., 2014). In some cases, such differences in targeting behavior are caused by the use of either homologous or heterologous systems for analysis (Fuss et al., 2013) but in most instances the reason remains enigmatic. Therefore, we have here systematically compared and evaluated the experimental *in vivo* approaches that are commonly used to study organellar protein transport in plants.

## Results

### Experimental set-up

For the comparative analysis of the most common *in vivo* approaches used to study intracellular protein targeting in plants, we have focused on four candidate proteins from *A. thaliana*, namely GCS (Glycine cleavage system subunit-H 1, At2g35370), GrpE (co-chaperone/nucleotide exchange factor GrpE I, At5g55200), EF-Tu (elongation factor Tu, At4g02930), and PDF (peptide deformylase 1B, At5g14660). While GCS, GrpE, and EF-Tu are considered to be mitochondrial proteins with confirmed functions in this organelle (Kuhlman and Palmer, 1995; Douce et al., 2001; Hu et al., 2012), PDF is addressed as plastid protein (Serero et al., 2001) showing additional import into mitochondria though (Giglione et al., 2000). The proteins were originally selected from a systematic *in silico* prediction approach and analyzed with respect to their targeting properties applying both *in organello* assays and transient transformation of pea epidermal leaf cells via particle bombardment. All four candidates demonstrated dual targeting properties in these assays (Baudisch et al., 2014). For comparison, two proteins with strictly monospecific organelle targeting characteristics (Rödiger et al., 2011) were analyzed in parallel, namely mtRi, the mitochondrial Rieske Fe/S protein from the respiratory electron transport chain of potato (GB:X79332.1) (Emmermann et al., 1994) and FNR (ferredoxin-NADP+-oxidoreductase, GB:M86349.1) of the photosynthetic machinery of spinach (Zhang et al., 2001).

For our analyses we have used reporter constructs as described in Baudisch et al. (2014), i.e., the N-terminal 100 amino acid residues of the respective precursor protein comprising the entire organelle targeting signal fused in-frame to eYFP (enhanced yellow fluorescent protein). The exception was FNR, where the defined transit peptide of 55 amino acids was instead fused to eGFP (enhanced green fluorescent protein). This was not possible for the candidate proteins because for them neither the exact processing sites nor the actual targeting signals have yet been characterized in detail. It is important to note that in all our assays identical constructs were used. Moreover, all these constructs showed targeting characteristics similar to those of the corresponding authentic precursor proteins when analyzed in *in organello* assays (Baudisch et al., 2014) demonstrating that in those cases the targeting behavior is not obviously affected by the reporter protein or the chimeric combination to a considerable extent. The constructs were cloned such that their expression in the plant cell is regulated by the CaMV 35S promoter and terminator.

### Particle bombardment

In the first set of *in vivo* experiments, we have transiently transformed the upper epidermis of *A. thaliana* leaves by particle bombardment. This assay leads to the transformation of only a few, isolated cells. Transformed cells are distinguishable from the surrounding, non-transformed tissue by the accumulation of the fluorescent reporter protein, which is detected using epifluorescence microscopy. One major advantage of this method is that it is suitable for the transformation of a wide variety of plant species (Klein et al., 1992; Chiu et al., 1996), thus allowing for the distinction between general and species-specific features of organelle targeting. For example, Van Aken et al. (2009) have shown that a candidate protein, GrpE, is targeted solely to mitochondria in *A. thaliana* cells, while dual targeting was observed in onion epidermal cells.

One disadvantage of this method is that it primarily transforms epidermal cells, which contain relatively small plastids with little amounts of chlorophyll (Barton et al., 2016). In those instances, where these plastids accumulate only low quantities of the fluorescent reporter protein, they are sometimes difficult to distinguish from mitochondria, which can mask vague fluorescence signals in plastids due to their high mobility and rapid fusion and fission (Supplementary Video S1).

**Figure 1:**
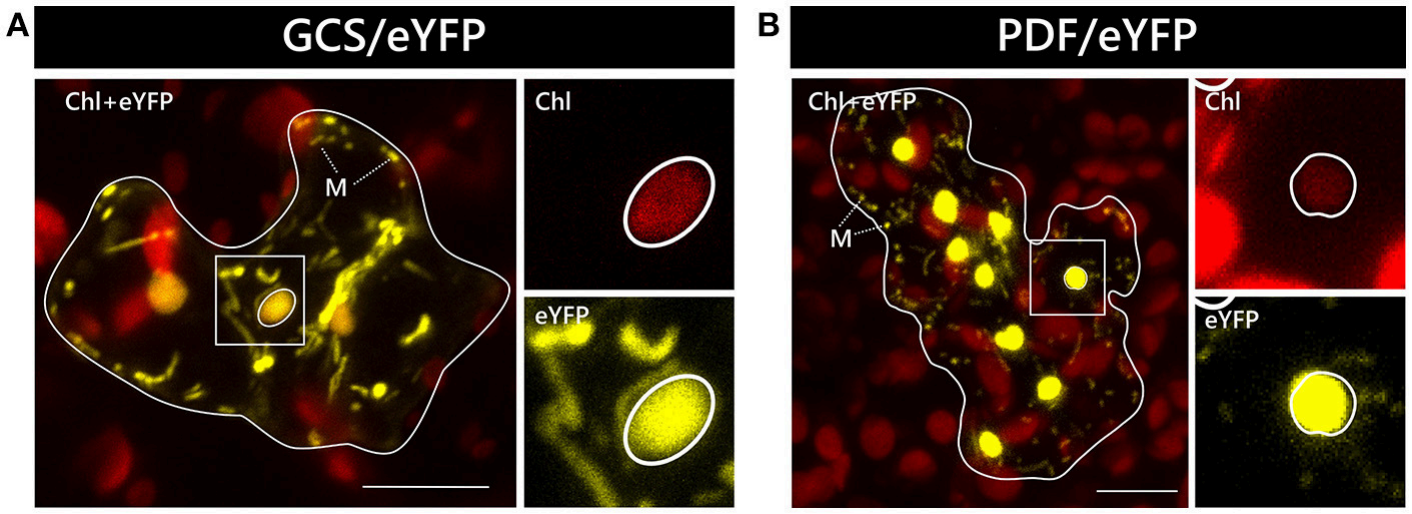
Dual localization of GCS/eYFP and PDF/eYFP after particle bombardment of *Arabidopsis thaliana* leaves. The coding sequences of GCS/eYFP **(A)** and PDF/eYFP **(B)** were transiently expressed under the control of the CaMV 35S promoter after particle bombardment of leaf epidermis cells of *Arabidopsis thaliana* and analyzed by confocal laser scanning microscopy. Representative cells showing dual localization of the candidate proteins in both mitochondria and chloroplasts are presented as overlay images of the chlorophyll channel (displayed in *red*) and the eYFP channel (displayed in *yellow*). The borders of the transformed cells are depicted by a continuous *white line*. The strong chlorophyll signals in the background are derived from the larger chloroplasts of untransformed mesophyll cells underneath the epidermal cell layers. The *squares* highlight areas of the transformed cells that are shown in higher magnification separately for the chlorophyll channel and the eYFP channel, as indicated. The position of representative plastids of each transformed cell is encircled for better visualization. Representative mitochondria are marked (*M*). The scale bars correspond to 10 μm.

**Figure 2:**
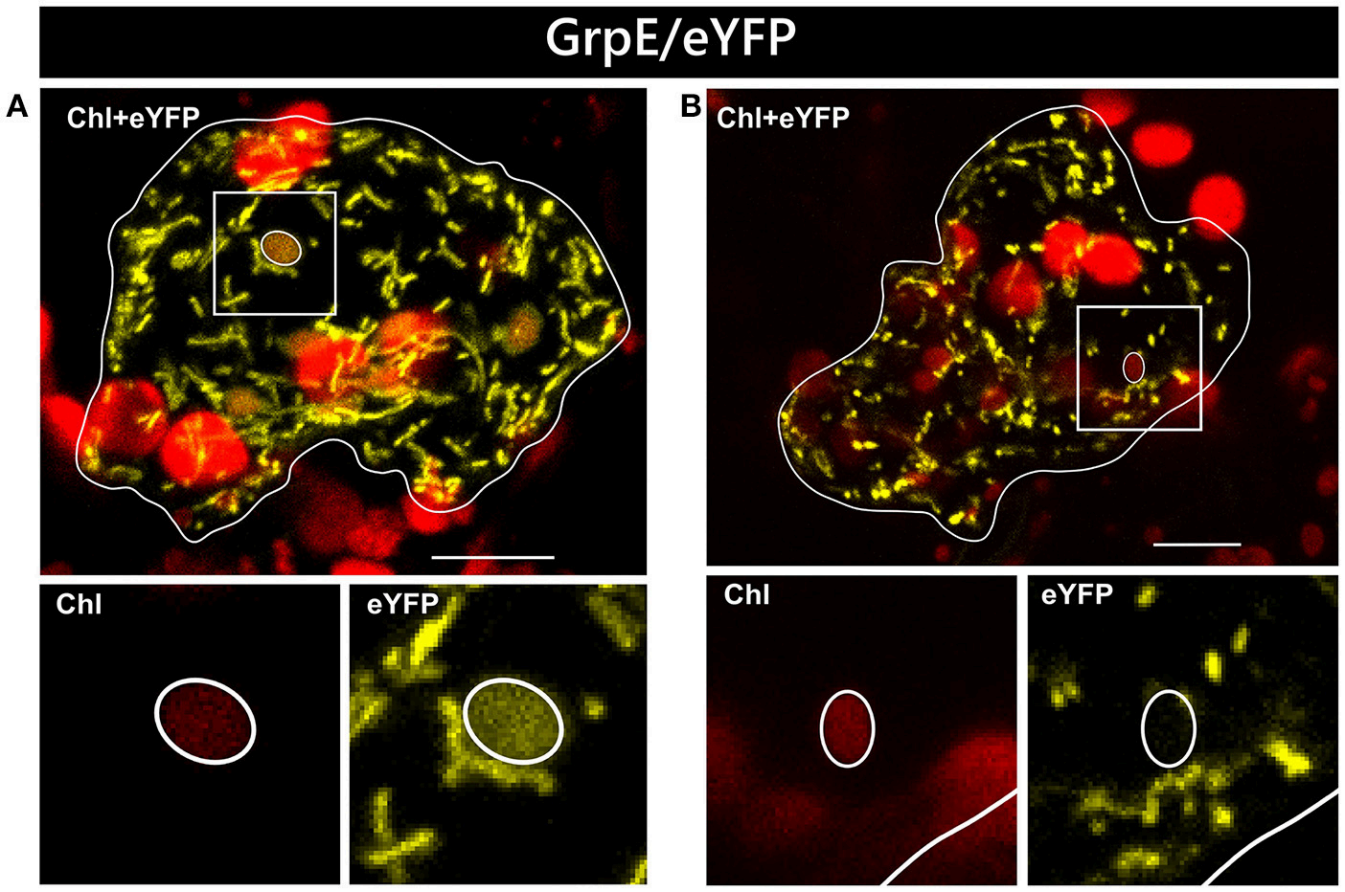
Differential localization of GrpE/eYFP after particle bombardment. Particle bombardment of leaf epidermis cells of *Arabidopsis thaliana* with the GrpE/eYFP construct can lead to either dual localization of the candidate protein in both mitochondria and chloroplasts **(A)** or to mitochondrial localization solely **(B)**. For further details see the legend of Figure 1.

**Figure 3:**
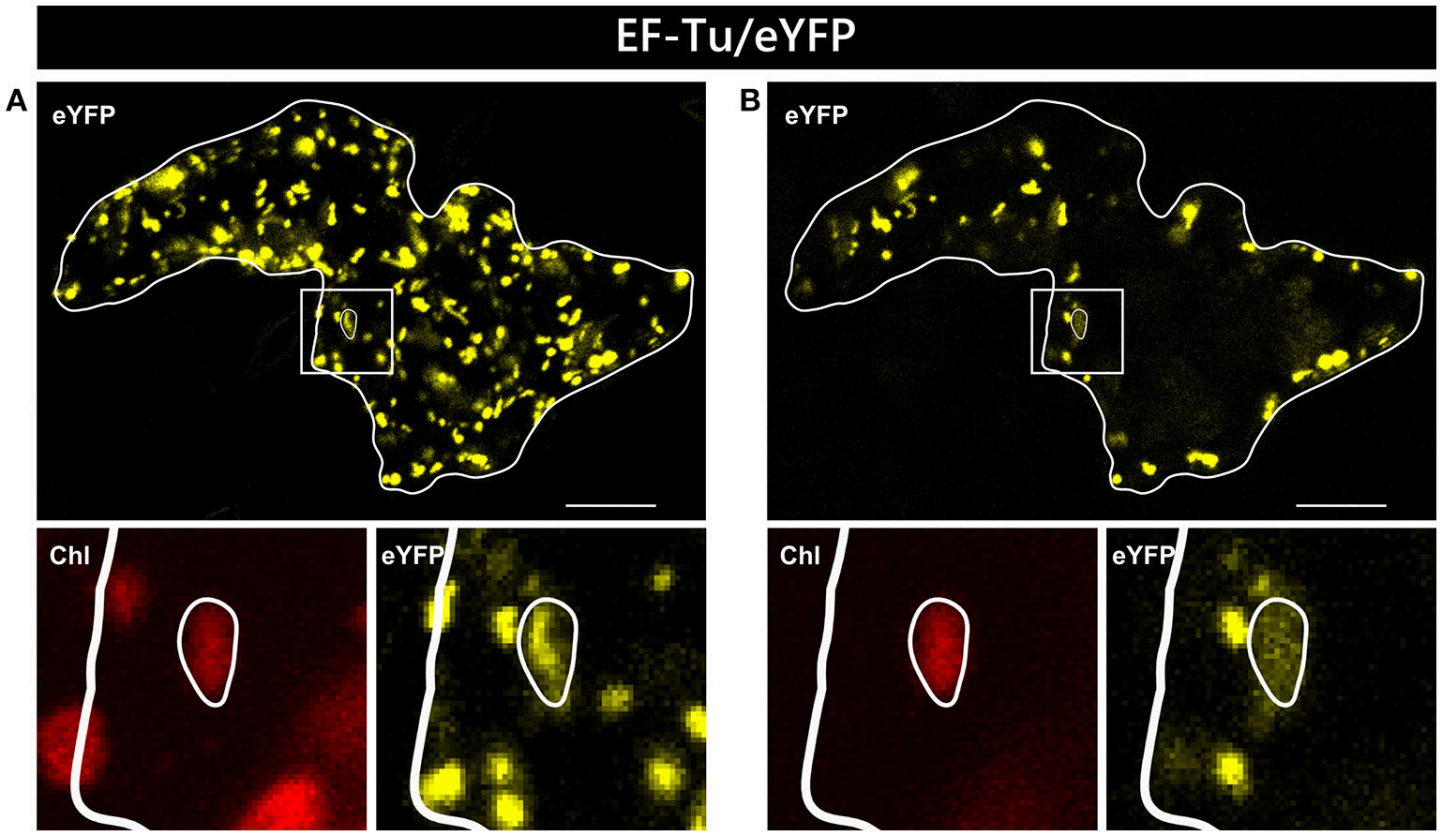
Alternative visualization of EF-Tu/eYFP localization in epidermal cells. The same epidermal cell of *Arabidopsis thaliana* transiently transformed by particle bombardment with the EF-Tu/eYFP construct is shown either as maximum intensity projection of several single images representing the complete cell in z-axis **(A)** or as a single plane image from the same acquisition **(B)**. In the areas below the overview pictures, separate images of eYFP and corresponding chlorophyll channels are shown at higher magnification. For further details see the legend of Figure 1.

In two independent experiments analyzing at least 10 cells each, only two candidate constructs, namely GCS/eYFP and PDF/eYFP, were dually targeted to both mitochondria and chloroplasts in all cases (Figure 1). In contrast, GrpE/eYFP showed differential targeting specificity in different cells. Within the very same experiment, GrpE/eYFP was dually targeted in some cells, but monospecifically targeted to mitochondria in others (Figure 2). This holds true also for EF-Tu/eYFP which was transported in all cells into mitochondria but only in few cases a faint fluorescence signal was detectable also in plastids. This is particularly well visible when single image planes are compared with complete Z-stack projections of the entire cell (Figure 3). And even these signals were difficult to visualize due to the considerable background resulting from the rapid movement of mitochondria, as mentioned above. Such cell-specific differential targeting behavior is not restricted to *A. thaliana* but could likewise be observed after particle bombardment of leaves from pea and *Nicotiana benthamiana* (data not shown). This suggests that, at least in these species, both endosymbiotic organelles can principally serve as targets for GrpE and EF-Tu. However, targeting behavior may be dictated by the physiological conditions present in individual cells.

For the two control proteins, namely FNR/eGFP and mtRi/eYFP, the expected monospecific targeting to plastids and mitochondria, respectively, was observed (Supplementary Figures S1A, S2A). In case of mtRi/eYFP, additional accumulation in the cytosol and nucleus was found in some cells (data not shown).

### Protoplast transformation

**Figure 4:**
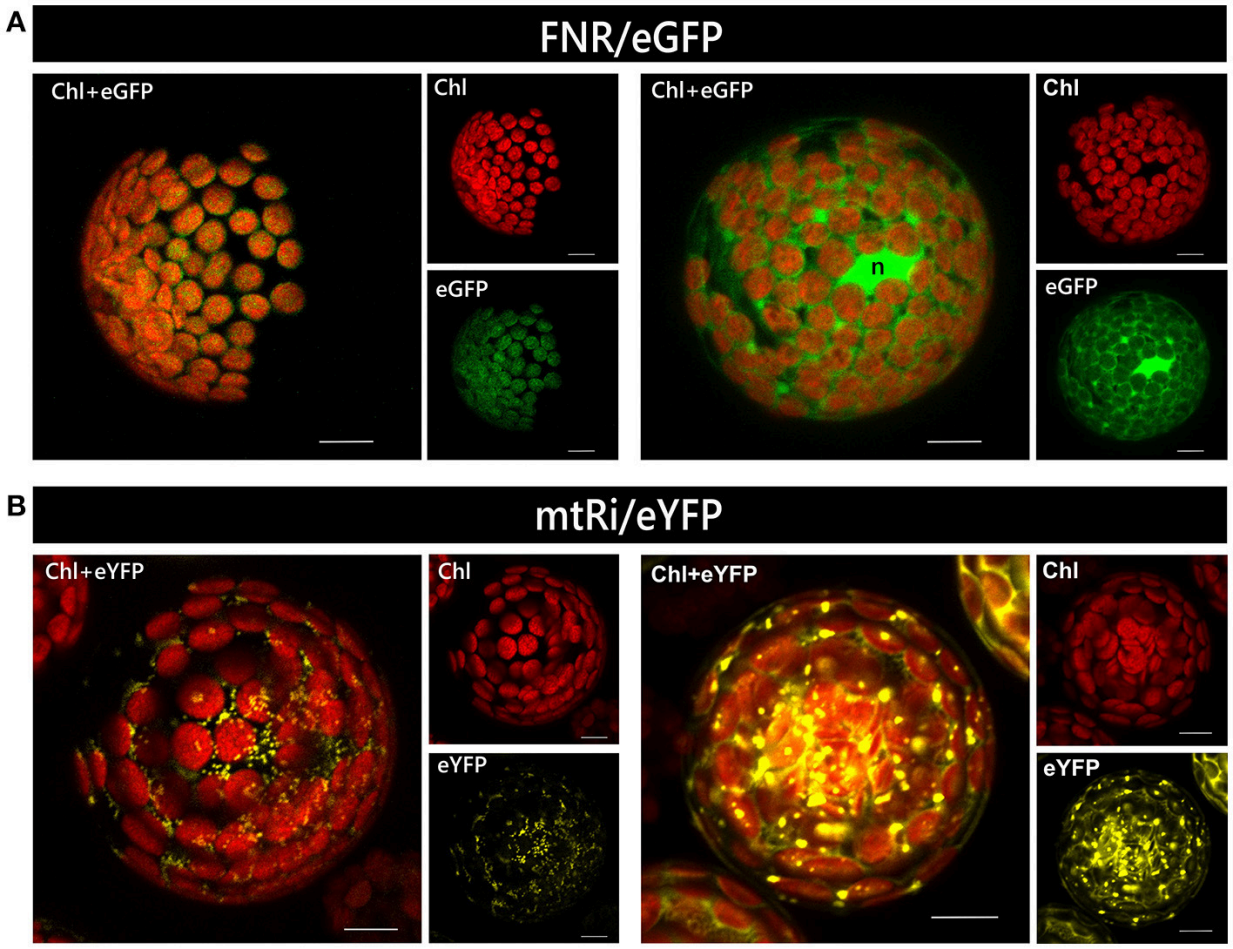
Relationship of expression rate and mislocalization in isolated protoplasts. Protoplasts isolated from leaves of *Arabidopsis thaliana* were transiently transformed with constructs encoding either FNR/eGFP **(A)** or mtRi/eYFP **(B)**, which usually leads to the accumulation of the reporter in chloroplasts and mitochondria, respectively (*left panels*). However, at high expression levels FNR/eGFP is often found predominantly in the cytosol rather than in plastids, while mtRi/eYFP is found accumulating as aggregates (*right panels*). All images are maximum intensity projections of several single images representing the complete protoplast in z-axis. The bright green area found in the right panel of image **(A)** probably represents the nucleus (*n*). For further details see the legend of Figure 1.

An alternative method for transient transformation, which is widely used and has likewise been established for many plant species, is protoplast transformation. It requires more efforts than particle bombardment but has the advantage that a large number of cells can be transformed simultaneously. The degree of gene expression, i.e., the amount of protein accumulating in the cell, varies substantially among different protoplasts of a single transformation assay because each of them represents an independent transformation event. Massive overexpression of a reporter construct, irrespective of the nature of its targeting signal, sometimes leads to artificial cytosolic localization, or even aggregation, of the protein, presumably due to saturation of the organellar import machinery (e.g., Figure 4).

In transformation experiments performed with protoplasts isolated from *A. thaliana*, all four candidate proteins showed dual targeting characteristics (Table 1). However, solely in the case of GCS/eYFP comparable fluorescence signals were observed in mitochondria and chloroplasts (Supplementary Figure S6B), while the other three candidates showed considerable differences in the signal intensities between the two organelles. For PDF, the *bona fide* plastid protein, the fluorescence intensity obtained with the reporter construct was less pronounced in mitochondria than in chloroplasts (Supplementary Figure S5B), whereas GrpE/eYFP and EF-Tu/eYFP showed reciprocal results, i.e., stronger fluorescence in mitochondria (Supplementary Figures S3B, S4B), in line with the results obtained after particle bombardment. For EF-Tu/eYFP, even exclusive mitochondrial localization was found in a few instances (Table 1).

**Table 1:** Localization of candidate proteins obtained with different experimental approaches.

	**Particle bombardment (*Arabidopsis*)**	**Protoplast transformation (*Arabidopsis*)**	**Agrobacterium infiltration (*Nicotiana*)**	**Transgenic plants (*Arabidopsis*)**	Mass spectrometry**(*Arabidopsis*)	**Literature data**
**GrpE/eYFP** (At5g55200*)	a) Dual b) Mito	**a) Dual**	a) Dual b) Mito	**a) Mito**	**Mito**	Dual (Onion)^a^ Mito (*Arabidopsis*)^a^ Dual (Pea)^b^
**EF-Tu/eYFP** (At4g02930*)	**a) Mito** b) Dual	a) Dual b) Mito c) Aggregates	a) Dual b) Mito	**a) Mito**	**Mito** Plastid	Dual (Pea)^b^
**GCS/eYFP** (At2g35370*)	**a) Dual**	**a) Dual** b) Aggregates	**a) Dual**	**a) Dual**	**Mito** **Plastid**	Dual (Pea)^b^
**PDF/eYFP** (At5g14660*)	**a) Dual**	**a) Dual**	**a) Dual**	**a) Dual**	**Plastid**	Dual (Pea)^b^ Dual (Onion)^c^
**mtRi/eYFP** (GB:X79332.1*)	**a) Mito** b) Cytosol + Nucleus	Mito Cytosol + Aggregates	**Mito** Cytosol	**a) Mito**	–	Mito (Pea)^b^
**FNR/eGFP** (GB:M86349.1*)	**a) Plastid**	a) Plastid b) Cytosol + Nucleus	**a) Plastid**	**a) Plastid**	–	Plastid (Pea)^b^

*Dual, localization in mitochondria and plastids; Mito/Plastid, localization exclusively in mitochondria/plastids; Aggregates, protein aggresomes in the transformed cell*.

a, b and c address distinct localization in different cells of the same experiment. Preferential or exclusive localization is highlighted in **bold**.

* Accession number of the corresponding candidate gene;

** Data obtained from MASCP GATOR (Joshi et al., 2011; Mann et al., 2013) and SUBA4 Databases (Hooper et al., 2017);

a (Van Aken et al., 2009);

b (Baudisch et al., 2014);

c *(Giglione et al., 2000)*.

### Agrobacterium infiltration of *nicotiana benthamiana*

The third transient transformation system studied here is an inexpensive and technically rather simple assay in which the lower epidermis of *N. benthamiana* leaves is infiltrated with cultures of *Agrobacterium tumefaciens* harboring the construct of interest. One advantage of this method is the large number of cells within an intact tissue that are simultaneously transformed in a single assay. The major disadvantage of this system is the fact that it is largely restricted to the host plant *N. benthamiana*, because *Agrobacterium-*mediated transient transformation is not as efficient in other plant species (Gelvin, 2003; Wroblewski et al., 2005). As a consequence, most candidate proteins are inevitably analyzed in a heterologous cell context if they originate from plant species other than *N. benthamiana*.

**Figure 5:**
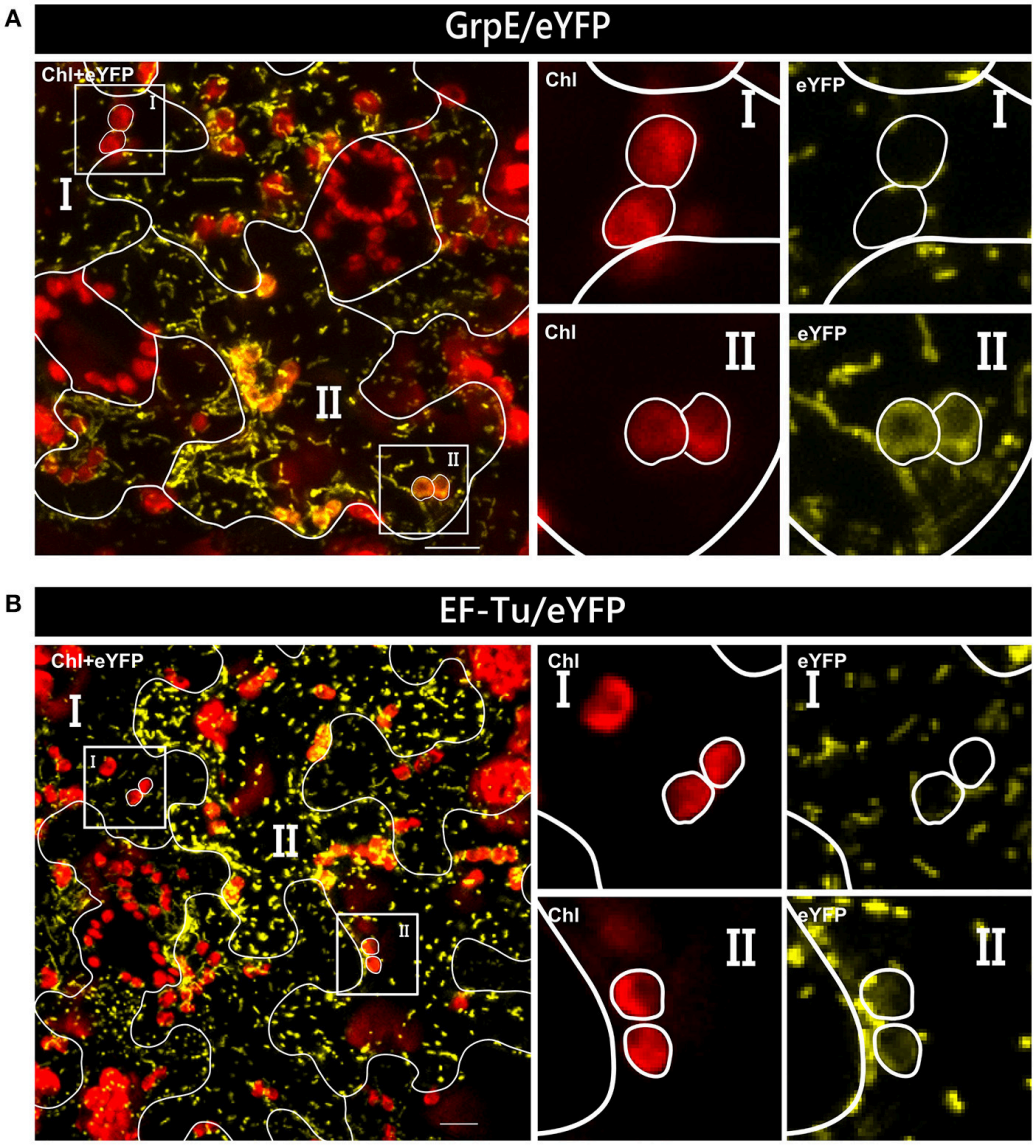
Variable organelle targeting in different cells of *Nicotiana benthamiana* after *Agrobacterium* infiltration. Confocal laser scanning microscopy of the lower epidermis of *Nicotiana benthamiana* infiltrated with *Agrobacterium tumefaciens* strain GV3101 carrying constructs encoding GrpE/eYFP **(A)** or EF-Tu/eYFP **(B)**. In both instances, dual targeting of the reporter protein is observed only in cells with strong expression of the candidate constructs (II), not in those with low expression levels (I), which show solely mitochondrial accumulation. For further details see the legend of Figure 1.

In our analyses, all four candidate proteins showed dual targeting into mitochondria and chloroplasts. However, not all cells are homogeneously transformed in such assays and patches of high and low intensity are usually visible in the transformed tissue. This difference in gene expression could influence even the transport behavior of a candidate protein. In the case of GrpE/eYFP and EF-Tu/eYFP, low expression of the reporter constructs leads to apparent monospecific targeting to mitochondria, since fluorescence signals could not be observed in plastids (Figure 5). Thus, there is a clear correlation of expression rate and targeting behavior which can be studied in such assays in a single step.

### Transgenic plants

Finally, stable transformation of *A. thaliana* using the floral-dip method was performed for comparison. This method is far more time-consuming than any of the transient transformation assays described above. On the other hand, it is assumed to yield the most reliable results because the candidate gene is usually integrated in only one or few copies into the nuclear genome, which should avoid major gene dosage effects. Still, even in this case different independent transgenic lines of each of our gene constructs showed considerably variable expression levels.

**Figure 6:**
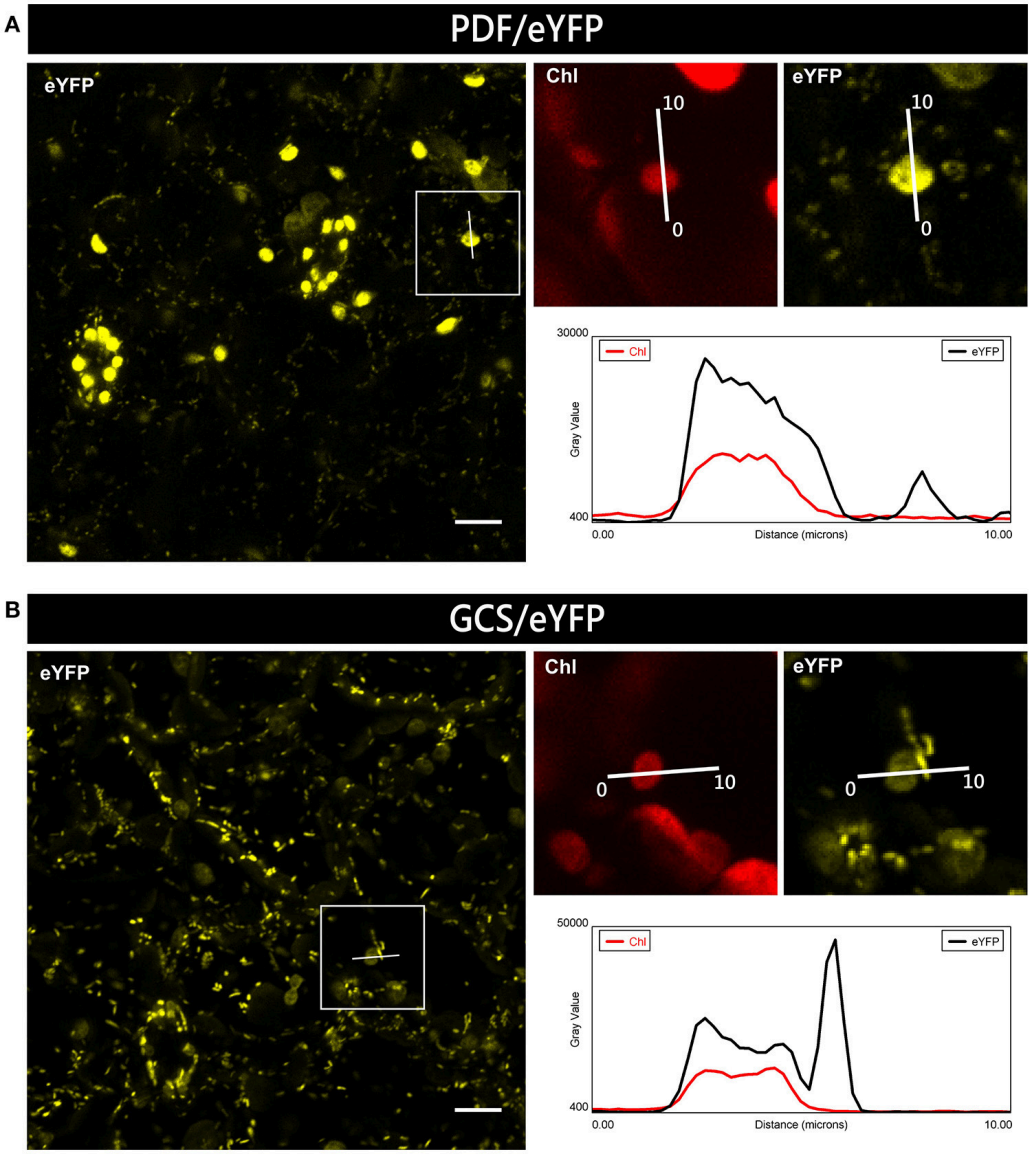
Preferential organellar accumulation of candidate proteins in transgenic *Arabidopsis* plants. Leaf epidermal cells of transgenic *Arabidopsis thaliana* plants constitutively expressing PDF/eYFP **(A)** or GCS/eYFP **(B)** show preferential accumulation of the fluorescent reporter protein in plastids and mitochondria, respectively. To compare the relative signal strengths in the two organelles, the degree of fluorescence was quantified using the plot profile tool of Fiji along a single line of 10 μm, covering in each case both a plastid and a mitochondrion. In the resulting graphs, *gray value* represents fluorescence intensity of the eYFP signal (*black line*) and the chlorophyll signal (*red line*). For further details see the legend of Figure 1.

For plants expressing PDF/eYFP or GCS/eYFP, clear dual targeting of the chimeric reporter protein was observed. However, the relative intensity of eYFP fluorescence for GCS/eYFP was higher in mitochondria, while PDF/eYFP showed stronger accumulation in plastids, which again indicates the potential preference of dually targeted proteins for one or the other organelle (Figure 6). Using a suitable image analysis program (e.g., Fiji software) such differences in targeting specificity can even be semiquantitatively analyzed.

In clear contrast to the results obtained with transient transformation, neither GrpE/eYFP nor EF-Tu/eYFP showed any chloroplast localization in the transgenic plants (Supplementary Figures S3D, S4D, respectively). Both proteins accumulated exclusively in the mitochondria of the analyzed leaf tissue. For EF-Tu/eYFP, the lack of chloroplast accumulation in the transgenic plants seems not surprising because the degree of plastid targeting was low in the transient assays, yielding only vague fluorescence signals (e.g., Figure 3). Considering a possibly mildly lower expression of this candidate gene in the transgenic lines, chloroplast transport would remain below the level of detection. In contrast, GrpE/eYFP showed clear dual targeting characteristics in all transient assays (Supplementary Figures S3A–C). Hence, in case of GrpE/eYFP, it appears that post-transport processes, like protein turnover rates, rather than incompetence of the transit peptide to mediate plastid import are responsible for the observed lack of chloroplast accumulation in the transgenic lines.

## Discussion

One remarkable outcome of the comparative evaluation of largely similar experimental systems utilized to determine the organellar targeting of nuclear-encoded proteins in plants is that divergent or even contradictory results can still all be valid and true (summarized in Table 1; for details see Supplementary Figures S1–S6). This appears impossible at first glance but may be explained by the fact that neither of the approaches reflects the organelle transport process in an unbiased manner. Instead, they all have their particular drawbacks and strengths, which become evident only if they are directly compared with each other.

### Transient vs. stable transformation

A good example for such variability is GrpE/eYFP which shows dual targeting in all transient assays described here but accumulates in transgenic plants solely in mitochondria (summarized in Supplementary Figure S3). At first glance this might suggest that all transient assays lead to artificial chloroplast import of the protein. However, this is probably a too simplistic view. Instead, the discrepancy observed after transient vs. stable transformation is probably a consequence of the different time-scales at which the analyses are performed. In transient assays, subcellular localization of the reporter construct is determined by fluorescence microscopy usually within 16–72 h after transformation, i.e., the protein is present in the cell for only a limited time period before being analyzed. In contrast, even if young tissue of transgenic plants is used for microscopy, the cells have expressed the reporter gene for several days or even weeks prior to analysis. Thus, regulatory processes, like protein turnover or counter selection caused by incompatibility of the reporter construct with the cell metabolism, can exert a major effect on the accumulation of the protein in the organelles. Regulating the time-point of transgene expression, for example by using inducible promoters, could circumvent such effects but this is not a common practice while studying protein targeting specificity.

A second point to be considered is the position at which the T-DNA carrying the candidate gene is inserted into the nuclear genome of the stably transformed plant line. In most instances, this insertion will take place in a non-essential region of the genome but it cannot be ruled out that occasionally also a gene is affected that plays a role in the subcellular targeting or accumulation of proteins. Consequently, it is essential to analyze more than a single transgenic line to prevent potential misinterpretation.

### Protoplasts vs. intact tissue

Even the various transient transformation systems have each their specific peculiarities. In transient transformation assays performed with intact tissue, like particle bombardment, single cells of known and defined origin are transformed. In contrast, isolated protoplasts always consist of a mixture of differentiated cell types. For example, protoplasts prepared from leaves of dicotyledonous plants will comprise not only mesophyll cells from palisade and spongy parenchyma but also epidermal and stomatal cells. Taking into account that protoplasts from different plant tissues can show differences in their protein targeting characteristics (Faraco et al., 2011), it can well be assumed that also unequally differentiated cells in the same assay will show different transport properties.

Furthermore, the procedure of protoplast isolation and transformation is stressful for the cell (Papadakis and Roubelakis-Angelakis, 2002). It has been reported that certain stress conditions can even lead to the release of proteins from organelles (for example plastids) resulting in their accumulation in the cytosol (Kwon et al., 2013). But maybe even more important is the fact that stress usually induces the expression of genes encoding chaperones like Hsp70 (Wang et al., 2004), which are involved also in protein import into mitochondria and chloroplasts (Zhang and Glaser, 2002). Together, these findings might explain why organelles can have different import characteristics in transient assays comparing protoplasts with cells of intact tissue.

### Presence or absence of *agrobacterium* in the assays

*Agrobacterium* infiltration of *N. benthamiana* usually yields a large number of transformed cells within an intact tissue that can be easily identified and analyzed at the subcellular level. This method therefore appears particularly suitable for large-scale protein localization studies. However, in addition to the fact that proteins analyzed are often of heterologous origin, the results may be confounded by symptoms resulting from the inoculation of the plant tissue with bacterial cells. In fact, it was shown that in leaf areas infiltrated with *A. tumefaciens*, defense reactions and chlorosis are sometimes induced (Pruss et al., 2008). Furthermore, depending on the *Agrobacterium* strain used for transformation, altered phytohormone levels in the plant tissue have been described (Erickson et al., 2014). At this point, it cannot be ruled out that such stress-related plant responses can well have an influence also on the organelle targeting of proteins. In fact, it is worthwhile to systematically analyze if and how such targeting processes depend on physiological conditions like the energy load of the cell or its redox status.

### Choice of the experimental system for protein transport studies

Considering the pros and cons of all experimental approaches described here, it becomes clear that there is no perfect method to study the specificity of protein targeting into organelles of intact plant cells. While the choice of the experimental system appears almost negligible in those cases in which a given candidate protein shows efficient transport with comparable rates into both endosymbiotic organelles (e.g., GCS/eYFP), it is much more important if the protein shows preferential targeting to one or the other organelle (e.g., EF-Tu/eYFP). In this case, more than a single approach is required to avoid misinterpretation, although it is obviously not a serious option to demand for all assays when analyzing a candidate protein.

Remarkably, even biochemical or proteomics approaches, which are often considered to be unbiased, have their specific inherent deficiencies. In principle, they depend strictly on the quality and, in particular, purity of the organelles studied. However, such extremely pure organelles cannot usually be obtained with standard isolation procedures and a certain degree of cross-contamination of, for example, mitochondria with proplastids is almost impossible to prevent (e.g., Keech et al., 2005; Rödiger et al., 2010). Furthermore, the methods of organelle isolation were usually established for “typical” organelles (e.g., mesophyll chloroplasts) and thus do not necessarily apply to other subtypes (e.g., epidermis chloroplasts) which can have deviating physico-chemical characteristics. Therefore, the data sets of organelle proteome analyses might well miss some organellar proteins but, on the other hand, inevitably comprise also a number of proteins from contaminating organelles. To cope with that, threshold levels are usually implemented to separate “true” from “wrong” results. However, such threshold levels are arbitrary and it might well be that a highly abundant contaminating protein shows an even higher value than an actual organelle protein of low abundance. This problem is particularly evident in the case of dually targeted proteins with preferential localization in only one of the two organelles and might be the reason for the sometimes discrepant proteomics data, e.g., for EF-Tu, which was found by mass spectroscopy always in mitochondria but only in a single case also in plastids (Helm et al., 2014; see also SUBA4 database).

In conclusion, the experimental system should be carefully chosen depending on the question to be answered, since each method addresses different aspects of the transport process. For example, if tissue specificity of the targeting process is anticipated, transgenic plants are the only valuable option. If instead targeting specificity in a homologous system needs to be studied, particle bombardment and/or protoplast transformation are the methods of choice. The latter cannot be applied though if stress needs to be avoided, which holds true also for *Agrobacterium* infiltration. In this case, particle bombardment or, even better, transgenic plants have to be utilized. And finally, if the principal property of a candidate protein to interact with the import machinery of an organelle is of interest, *in organello* import experiments performed with isolated intact organelles are still an option.

## Materials and methods

### Reporter constructions

The reporter constructs used for particle bombardment are based on vector pRT100mod (pRT100 Ω/Not/Asc; Überlacker and Werr, 1996) and have been described in Baudisch et al. (2014). For subsequent cloning into the binary vector pCB302 (Xiang et al., 1999), the entire chimeric genes including CaMV 35S promoter and terminator (constructs GCS/eYFP, GrpE/eYFP, EF-Tu/eYFP, and mtRi/eYFP) were recovered after digestion with *Sda*I and ligated into pCB302 digested with PstI. Reporter constructs PDF/eYFP and FNR/eGFP were instead inserted into the binary vector pLSU4GG (Erickson et al., 2017) using Golden Gate cloning. All binary constructs were subsequently used to transform *A. tumefaciens* strain GV3101.

### Particle bombardment

Particle bombardment of 3–5 weeks old leaves from *A. thaliana Col-0* plants was performed as described (Rödiger et al., 2011). For each bombardment, 300 ng plasmid DNA were precipitated with 2.5 M CaCl_2_, 0.1 M Spermidin onto 0.2 mg Gold particles (0.6 μm, Bio-Rad). Leaves were bombarded on the adaxial side and incubated for 16–20 h in the dark prior to microscopic analysis.

### *Agrobacterium* infiltration

*Agrobacterium* strains carrying the candidate gene constructs were harvested after incubation for 72 h at 28°C from LB agar plates supplemented with the appropriate antibiotic. After resuspension in infiltration medium (10 mM MgCl_2_, 10 mM MES, 150 μM acetosyringone) the cultures were adjusted to OD_600_ = 0.8, incubated for 3 h at room temperature and infiltrated with a needleless syringe into the lower epidermis of fully expanded leaves from 6 to 8 weeks old *N. benthamiana* plants. After incubation for 3 days with a 16/8 h light-dark cycle, protein localization was analyzed using confocal laser scanning microscopy (CLSM).

### Protoplast isolation and transformation

Protoplasts were isolated following the “Tape *Arabidopsis* sandwich” method and transformed as described (Wu et al., 2009) except that 10 μg plasmid DNA was used in each transformation assay.

### Generation of transgenic *arabidopsis* lines

Wild-type *A. thaliana Col-0* plants were transformed using the floral dip transformation method (Davis et al., 2009). Transformed plants were selected by either spraying with 0.1% BASTA (pCB302 constructs) or on $\frac{1}{2}$ MS plates supplemented with 30 μg/ml Hygromycin-B (pLSU4GG constructs). In all instances at least three independent T1 and T2 transgenic lines each were analyzed by confocal microscopy.

### Confocal microscopy and image processing

Confocal laser scanning microscopy was carried out with a Zeiss LSM780 Confocal Imaging System. For the emission of fluorescence signals specimens were excited with either 488 nm (eGFP), 514 nm (eYFP) or 633 nm (chlorophyll), usually with 2% of full laser power. Images were collected using filters ranging from 493 to 598 nm (eGFP), 519–620 nm (eYFP), or 647–721 nm (chlorophyll). Image acquisition was done in several Z-stacks and presented as maximum intensity projection. Essentially identical settings were used for acquisition of all images. Brightness and contrast of the images were later adjusted in order to better visualize the fluorescence signals. All images were processed using ZEN software (Carl Zeiss, Jena) and Inkscape^TM^ (GPL, v3). Quantification of the signals was performed with raw images using the Fiji program (Schindelin et al., 2012).

## Author contributions

MS performed the experiments. MS, BB, and RK designed the project and wrote the manuscript. RK supervised the study.

### Conflict of interest statement

The authors declare that the research was conducted in the absence of any commercial or financial relationships that could be construed as a potential conflict of interest.
