# Supplementary material for: Dual or Not Dual?—Comparative Analysis of Fluorescence Microscopy-Based Approaches to Study Organelle Targeting Specificity of Nuclear-Encoded Plant Proteins
Source: Front Plant Sci. 2018 Sep 19;9:1350. doi: 10.3389/fpls.2018.01350 (PMC6160753; doi:10.3389/fpls.2018.01350)

Supplementary Material

Dual or not dual? - Comparative analysis of fluorescence microscopy-based approaches to study organelle targeting specificity of nuclear-encoded proteins

Mayank Sharma, Bationa Bennewitz, and Ralf Bernd Klösgen*

*** Correspondence:**

Ralf Bernd Klösgen: klosgen@pflanzenphys.uni-halle.de

Supplementary Figures S1 - S6.

Comparison of the subcellular localization of candidate proteins in different experimental systems. Subcellular localization of the candidate proteins FNR/eGFP (suppl. Fig. S1), mtRi/eYFP (suppl. Fig. S2), GrpE/eYFP (suppl. Fig. S3), EF-Tu/eYFP (suppl. Fig. S4), PDF/eYFP (suppl. Fig. S5), and GCS/eYFP (suppl. Fig. S6), as determined by confocal laser scanning microscopy after biolistic transformation of leaf epidermal cells of *Arabidopsis thaliana* (panels a), protoplast transformation of *Arabidopsis thaliana* (panels b), *Agrobacterium* infiltration of *Nicotiana benthamiana* (panels c), and in transgenic *Arabidopsis thaliana* lines (panels d). All images are maximum intensity projections of several single images representing the complete cell in z-axis. Overlay pictures of both the eYFP channel (displayed in yellow) and the chlorophyll channel (displayed in red) are shown. Separate images of the two channels are shown at higher magnification as insets. The scale bars correspond to 10 µm.

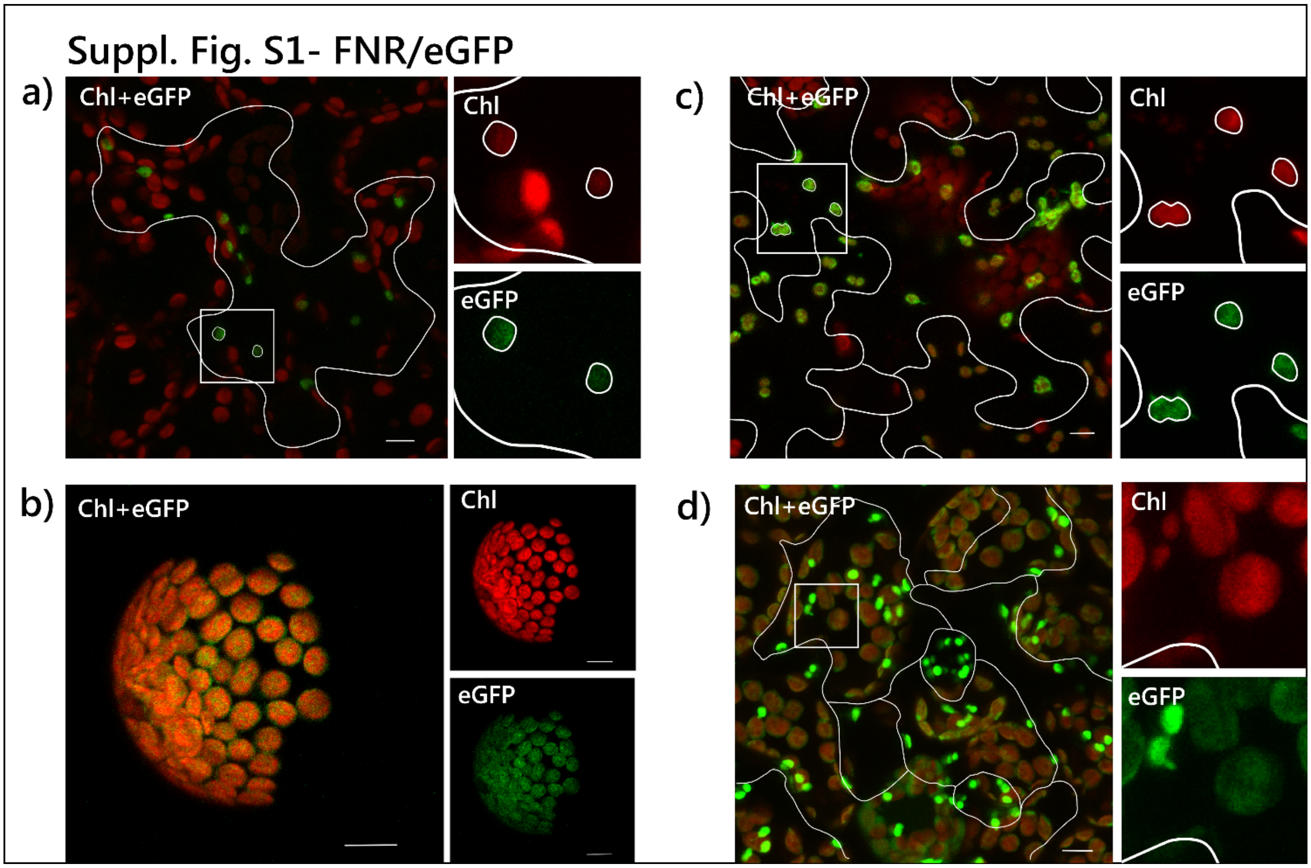
Suppl. Fig. S1- FNR/eGFP
a)
Chl+eGFP
Chl
eGFP
b)
Chl+eGFP
Chl
eGFP
c)
Chl+eGFP
Chl
eGFP
d)
Chl+eGFP
Chl
eGFP

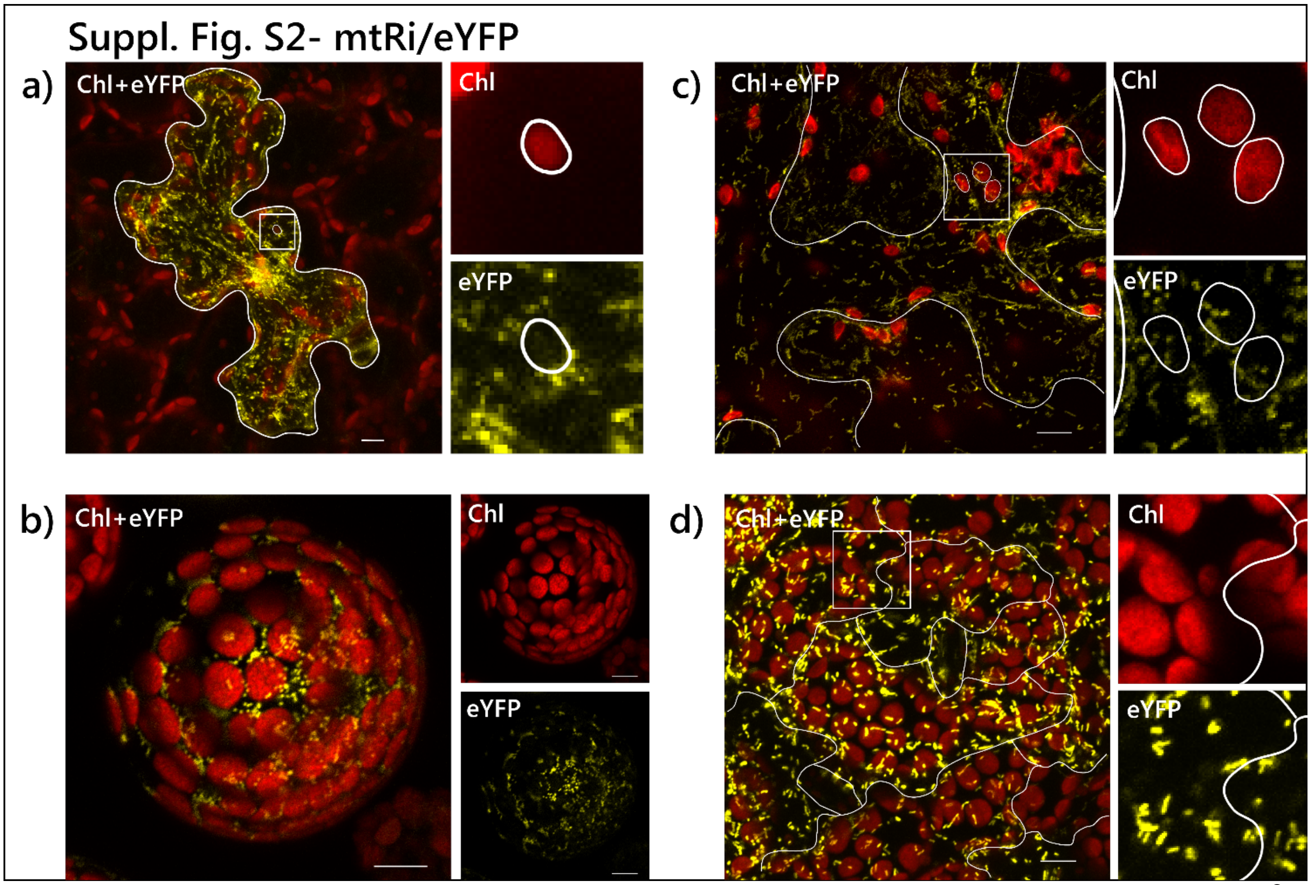
Suppl. Fig. S2- mtRi/eYFP
a)
Chl+eYFP
Chl
eYFP
b)
Chl+eYFP
Chl
eYFP
c)
Chl+eYFP
Chl
eYFP
d)
Chl+eYFP
Chl
eYFP

Suppl. Fig. S3- GrpE/eYFP

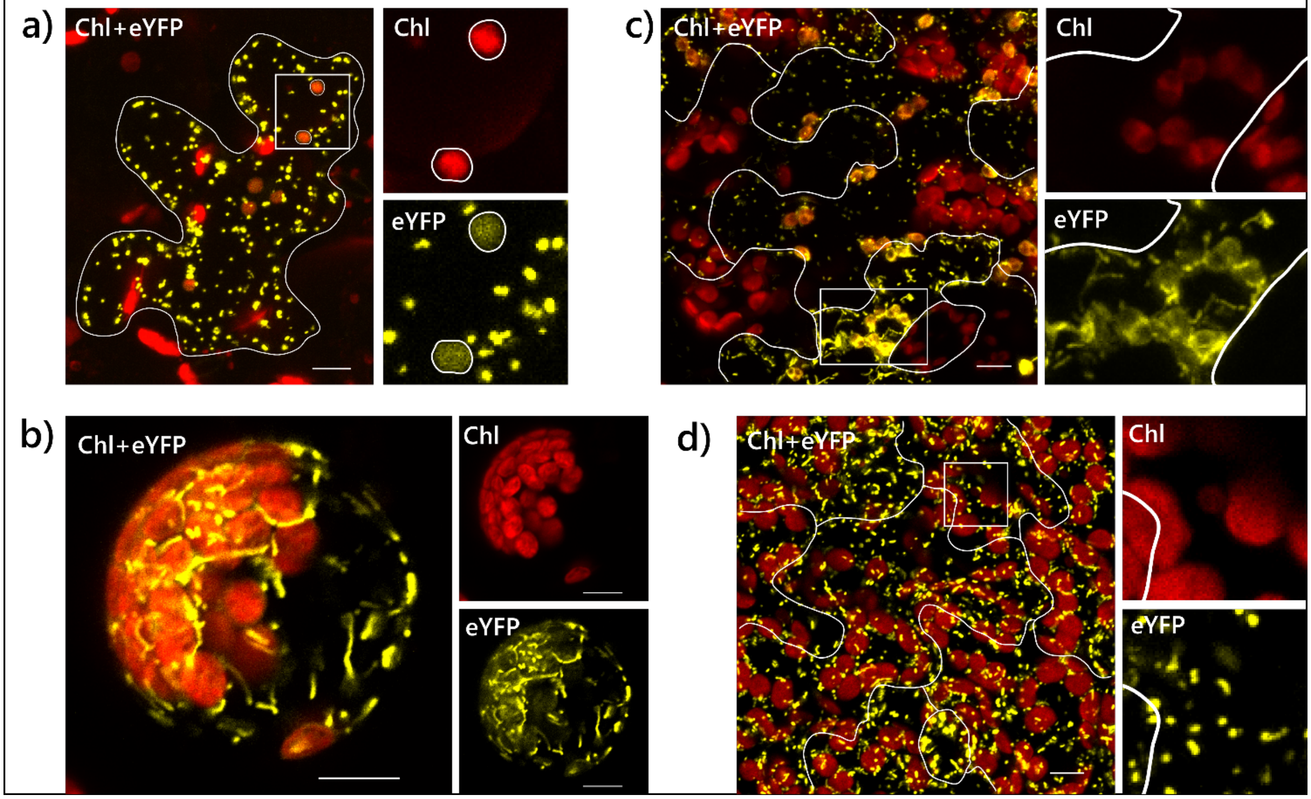

Suppl. Fig. S4- EF-Tu/eYFP

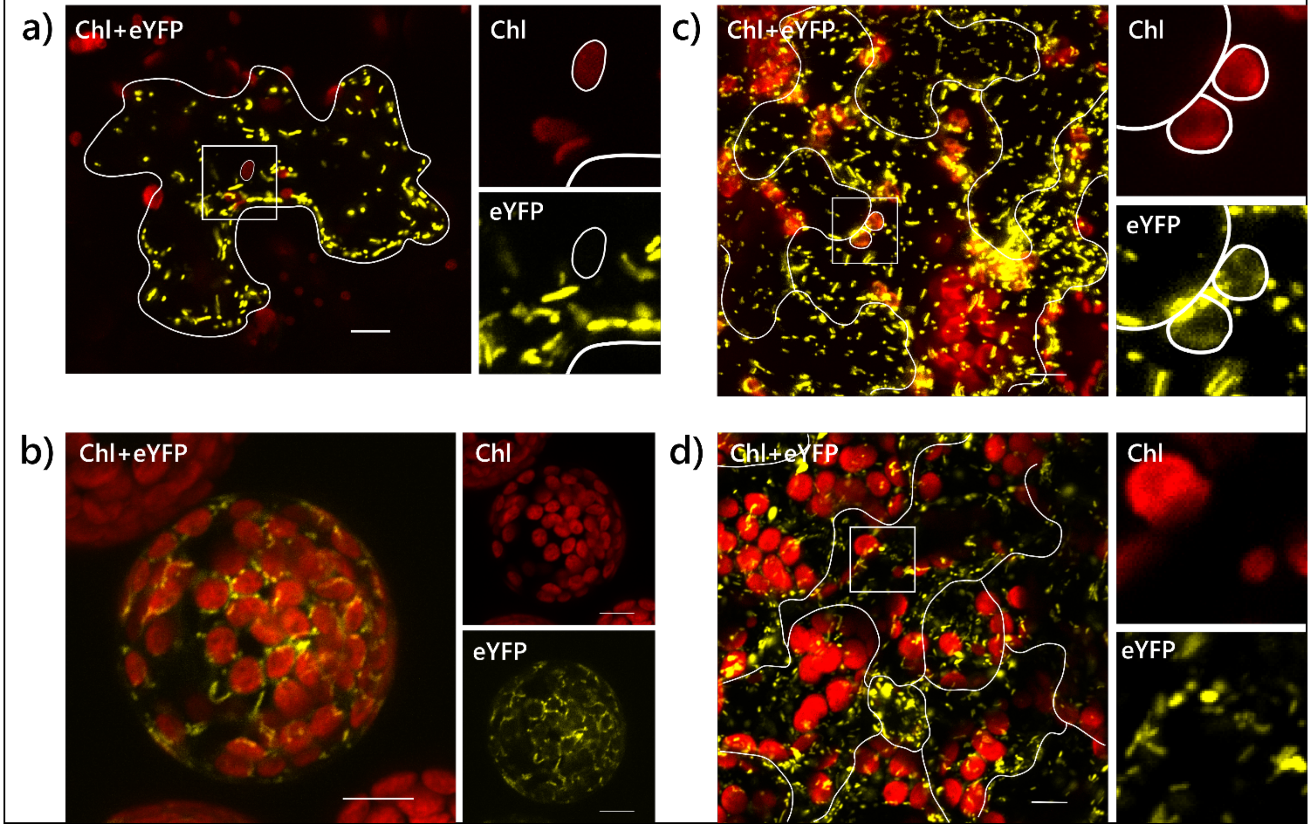

Suppl. Fig. S5- PDF/eYFP

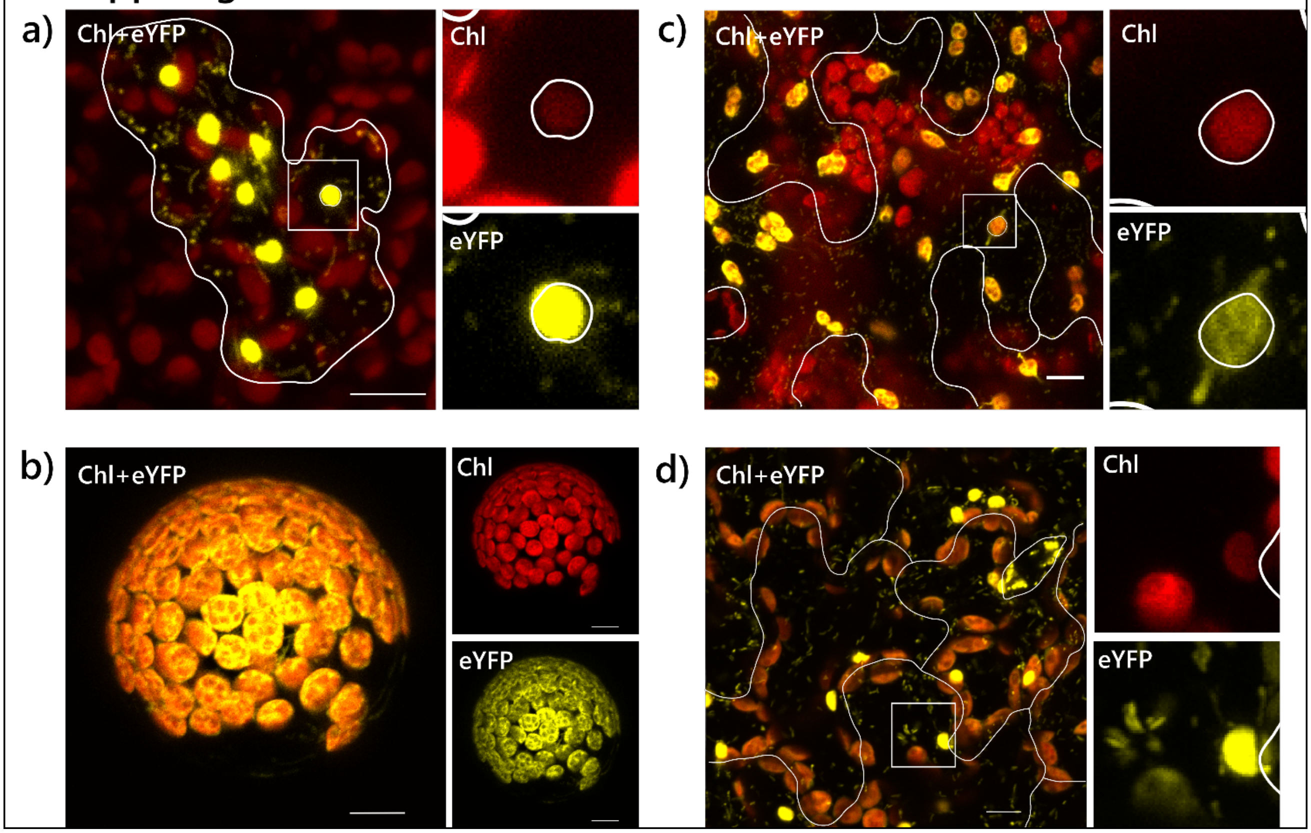

Suppl. Fig. S6- GCS/eYFP

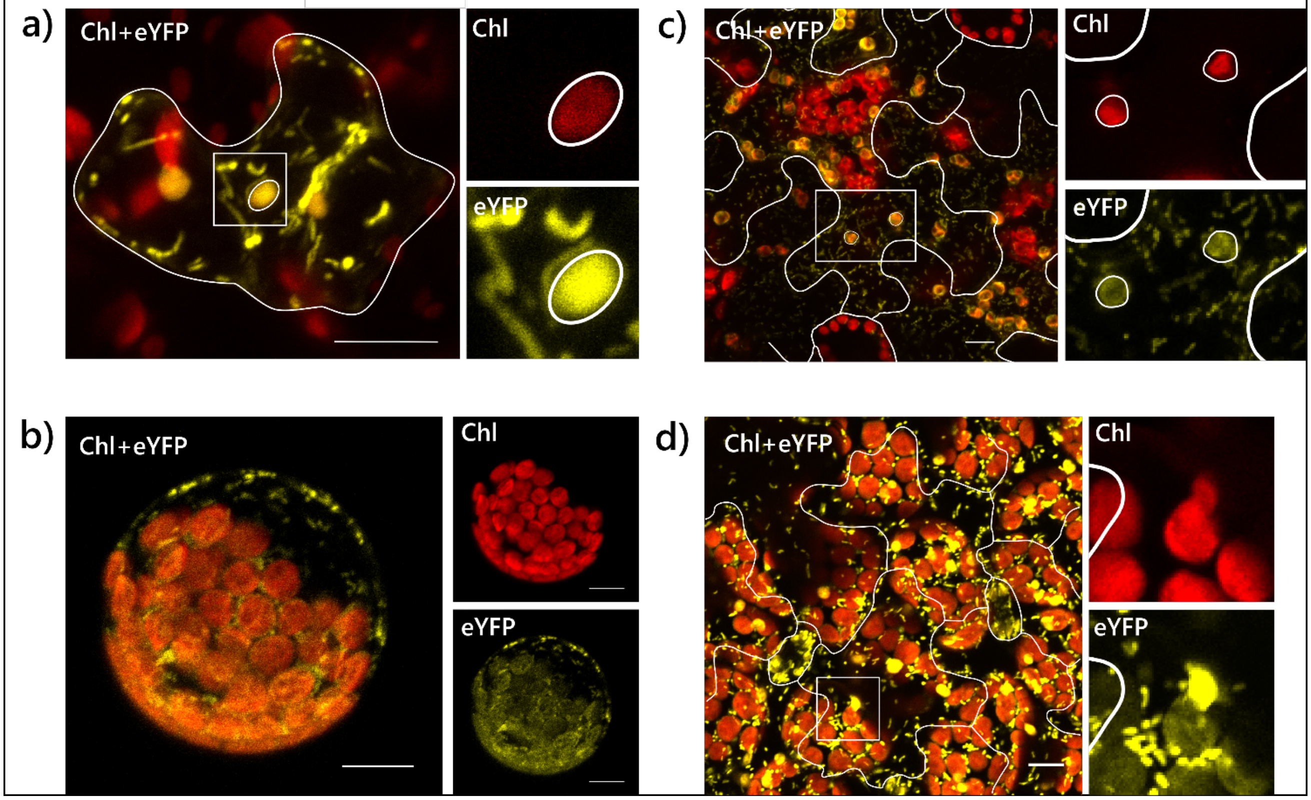

Supplement: Supplementary file 2 [file Image_1.PDF]
